# Predicting 28-day compressive strength of fibre-reinforced self-compacting concrete (FR-SCC) using MEP and GEP

**DOI:** 10.1038/s41598-024-65905-5

**Published:** 2024-07-27

**Authors:** Waleed Bin Inqiad, Muhammad Shahid Siddique, Mujahid Ali, Taoufik Najeh

**Affiliations:** 1 Military College of Engineering (MCE), National University of Science and Technology (NUST), Islamabad, 44000 Pakistan; 2 Department of Transport Systems, Traffic Engineering and Logistics, Faculty of Transport and Aviation Engineering, Silesian University of Technology, Krasińskiego 8 Street, 40-019 Katowice, Poland; 3 Operation and Maintenance, Operation, Maintenance and Acoustics, Department of Civil, Environmental and Natural Resources Engineering, Lulea University of Technology, Lulea, Sweden

**Keywords:** Self-compacting concrete, Genetic Programming, Fiber-reinforced self-compacting concrete, Multi expression programming, Gene expression programming, Engineering, Civil engineering

## Abstract

The utilization of Self-compacting Concrete (SCC) has escalated worldwide due to its superior properties in comparison to normal concrete such as compaction without vibration, increased flowability and segregation resistance. Various other desirable properties like ductile behaviour, increased strain capacity and tensile strength etc. can be imparted to SCC by incorporation of fibres. Thus, this study presents a novel approach to predict 28-day compressive strength (C–S) of FR-SCC using Gene Expression Programming (GEP) and Multi Expression Programming (MEP) for fostering its widespread use in the industry. For this purpose, a dataset had been compiled from internationally published literature having six input parameters including water-to-cement ratio, silica fume, fine aggregate, coarse aggregate, fibre, and superplasticizer. The predictive abilities of developed algorithms were assessed using error metrices like mean absolute error (MAE), a20-index, and objective function (OF) etc. The comparison of MEP and GEP models indicated that GEP gave a simple equation having lesser errors than MEP. The OF value of GEP was 0.029 compared to 0.031 of MEP. Thus, sensitivity analysis was performed on GEP model. The models were also checked using some external validation checks which also verified that MEP and GEP equations can be used to forecast the strength of FR-SCC for practical uses.

## Introduction

The manufacture of cement prompts ${\text{CO}}_{2}$ emission in atmosphere and depletion of natural resources like limestone etc. The annually manufactured quantity of cement is around 4000 million tons^1^ and it is the most emission intensive substance from the construction industry which contributes 7% to the global carbon emissions alone^2^. The release of these harmful greenhouse gases in atmosphere poses a serious threat to humanity and result in rising global temperatures, melting of glaciers etc. To reduce the carbon emissions associated with production of cement, new materials are being introduced with the aim to replace cement in concrete^3^. These products include a variety of materials like marble powder, fly ash, silica fume, slag etc. and are known as Secondary Cementitious Materials (SCMs). The utilization of these SCMs in concrete can help to lessen the ill effects of concrete industry on the environment for a sustainable future^4^. The recent advancements in low-carbon concrete composites have led to the invention of a new form of concrete named as SCC. It was invented in Japan in 1980s^5^. Soon after its development, it found many uses in buildings, bridges, and precast concrete components around the world^6^ due to its advantages such as high structural quality, increased productivity, and durability^7^. SCC has many properties that deviate from that of normal concrete. The most important being its ability to flow and compact itself under its own weight. It can flow and fill the spaces in members that have congested arrangement of reinforcement or where conventional vibrating methods are not applicable^8^. SCC also offers many other advantages such as segregation resistance, improved surface finish, and increased durability. It also leads to improvement in the site conditions by eliminating the need of mechanical compaction methods such as vibrators that cause fossil fuels burning and ${\text{CO}}_{2}$ emission^9^.

### Fibre-reinforced SCC

**Figure 1:**
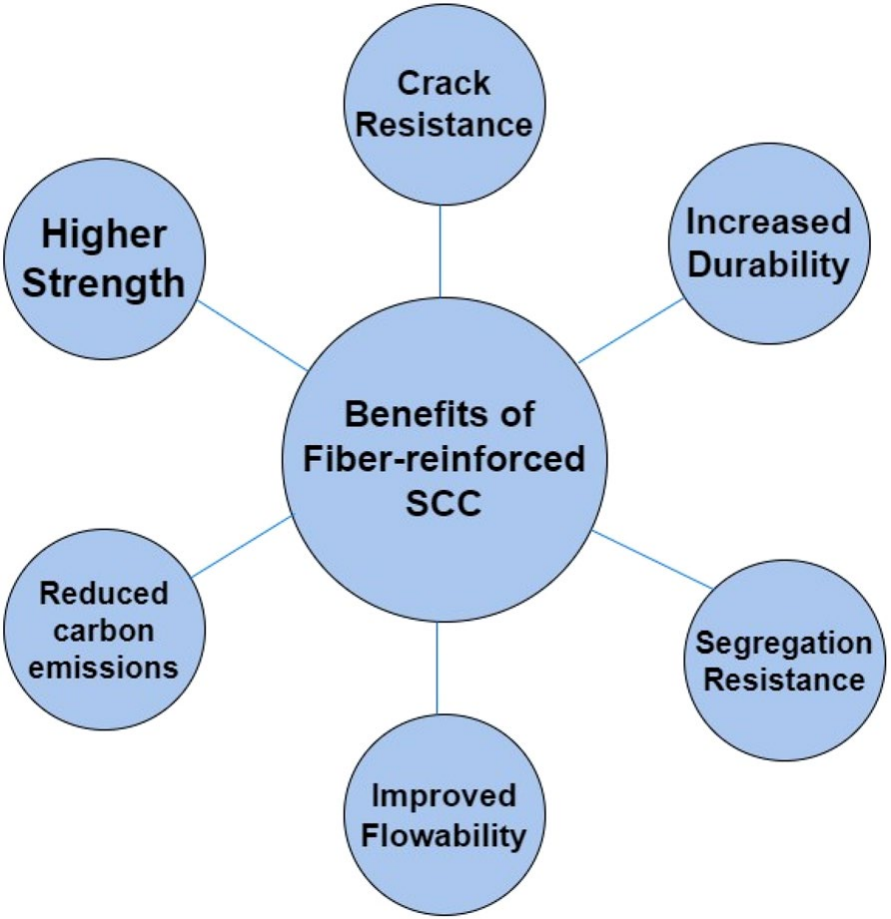
Benefits of fibre-reinforced SCC.

The SCC mix design is at the heart of achieving the desirable characteristics. Generally, SCC requires higher water-to-cement ratio and the use of chemical admixtures to impart the flowability characteristics to SCC. Also, the mix design calls for the use of greater quantity of fine materials to fill the spaces between coarse aggregate^10^. Since the use of huge quantities of cement to increase the level of fines can be costly, researchers have suggested using several mineral admixtures as a replacement of cement in SCC^11–14^. The use of these admixtures results in cost reduction and improve strength and durability characteristics^15–20^. Despite the benefits of SCC as an innovative material, it has the drawback of being a brittle material having low tensile strength and strain capacity like normal concrete^21,22^. Thus, the incorporation of fibres is desirable to render a ductile behaviour to SCC and improve its crack resistance, tensile strength, and strain capacity^23^. Fibre-reinforced concrete is created by adding short and discontinuous fibres that are dispersed throughout the concrete matrix^24^. The various types of fibres used in industry include steel fibres, glass fibres, polypropylene fibres etc. It has been reported that using a mix of fibres significantly improves the concrete properties^25^. Thus, this study used a dataset collected from literature having a mix of polypropylene and glass fibres. The incorporation of fibres in SCC results in the development of F-SCC which results in improvement of post-cracking behaviour by preventing the propagation of small and newly developed cracks^23^.Thus, F-SCC offers many added advantages compared to non-reinforced SCC as shown in Fig. 1. However, for effective utilization of F-SCC in construction industry, we must be able to accurately predict its 28-day compressive strength because it is an indicator of overall concrete quality and durability. However, to effectively utilize a revolutionary product like FR-SCC we must be able to accurately estimate its different properties out of which 28-day compressive strength (C–S) is the most important one. The 28-day compressive strength test is useful to check the overall quality and performance of concrete^26–29^. But it has been observed that a comprehensive experimental investigation to check the mechanical properties of new concrete composites is time consuming as a large number of loading conditions, exposure types and mixture compositions must be studied to get an accurate estimate of the properties^30^. Also, it is not an easy task to conduct these experiments in identical and controlled environment due to the inherent complexities and limitations of the conventional testing procedures^31^. Moreover, most of the standard tests are destructive in nature means that the specimen is destroyed during the test, so this also contributes to the resource wastage and increases construction waste. Thus, there are many limitations to the testing of concrete composites using conventional testing methods.

To overcome the limitations stated above, researchers have come up with the idea that mathematical models should be used to predict the different properties of composite materials^32^. These machine learning models reduce the time, effort, and cost required for experimental investigation and accurately predict the behaviour of different materials under various conditions without conducting experiments^33^. The literature regarding the utilization of several famous machine learning techniques to predict different concrete properties is described in next section. In Section “Research significance”, the particular research novelty and significance has been highlighted followed by detailed explanation of the employed algorithms in Section “Research methodology”. Section “Research methodology” also sheds light on data collection which was used to build the predictive models and statistical analysis performed on the data while Section “Results and discussions” deals with the results of the algorithms.

### Overview of machine learning in civil engineering

During the last few years, civil engineering industry is making a shift towards the sustainable materials and data-driven decision-making tools like every other industry. In the field of civil engineering, AI is mainly used to optimize material utilization, foster sustainable construction, and improve the overall efficiency and accuracy of construction processes by using various machine learning and deep learning techniques. Machine learning (ML) is a subset of artificial intelligence (AI) which refers to the process in which machines learn from vast amounts of data without any human intervention. ML techniques learn patterns from the data and use that to make informed decisions for future data. Deep learning is in turn a subset of ML which refers to the use of neural networks to make predictions on unseen data. Recently, the prediction of various properties of new concrete composites using machine learning (ML) models has captured the interest of researchers. This is due to the simplicity, accuracy, cost, and time effectiveness of the ML techniques^32^. ML algorithms are basically computational methods designed to solve complex problems like humans. It has been reported that ML algorithms can discover the hidden patterns in the data and make predictions based on the learning from the data^34^. ML techniques have been successfully applied to solve problems related to finance, biology, engineering etc. In the domain of civil engineering, ML techniques have found their use in predicting various properties of different concrete and cement composites^35–40^, composite concrete columns and tubes^41–45^, soil classification and compaction factors etc.^46,47^. ML techniques have also found their uses in geotechnical and mining engineering to determine slope stability, landslide susceptibility, and rock fracture prediction etc.^48–60^. Moreover, ML algorithms have been used to determine service life of concrete structures in marine environments^61^ and health monitoring of asphalt pavements^62^.

## Literature review

The prediction of SCC properties using various ML algorithms like Support Vector Machine (SVM), Artificial Neural Networks (ANN), etc. has increased in the last few years due to the immense potential of SCC to reduce the concrete associated carbon emissions. In 2021, Farooq et al.^63^ utilized different ML algorithms including ANN, SVM and GEP to predict CS of fly ash containing SCC. The study concluded that GEP algorithm is the accurate one having average error of only 3.71 followed by 5.03 and 5.42 for ANN and SVM respectively. Similarly in 2022, Abunassar et al.^64^ utilized ANN and SVM to predict CS of SCC having silica fume and fly ash as admixtures. The authors utilized 85 data points to develop ML models that have seven input and one output variable. The comparison was drawn between the two algorithms based on the ${\text{R}}^{2}$ value when models were used to predict unseen test data. The results showed that both techniques are reliable and accurate to predict CS of SCC, but SVM achieved ${\text{R}}^{2}=0.97$ compared to 0.96 of ANN thus it is more accurate than ANN. Also, Asteris et al.^10^ utilized ANN to forecast strength of SCC having a wide variety of admixtures. These admixtures included slag, fly ash, limestone powder, silica fume, rice husk ash, etc. The employed a dataset of 169 points and the accuracy of resulting model was measured using coefficient of determination. The authors concluded that ANN showcased a great ability to predict strength of SCC blended with various admixtures having ${\text{R}}^{2}$ value equal to 0.98. In the same way, the strength estimation of lightweight concrete made by using oil palm by-product and concrete made with pozzolanic admixtures was done by^65^. Moreover, to foster the utilization of recycled construction waste in concrete, Ashrafian et al. utilized neuro-fuzzy algorithm to provide accurate prediction model for compressive strength of environmentally friendly concrete containing recycled construction wastes^66^. The authors gathered an extensive database of more than 300 concrete mixture proportions having construction waste and used a neuro-fuzzy algorithm with a Horse Herd Optimization Algorithm (HOA) for the purpose of strength estimation and reported that this approach performed well than other standalone algorithms and optimization approaches for prediction of strength.

As far as the subject of prediction of F-SCC properties is concerned, there are very few studies available. The important ones include the study conducted by Rahman et al.^33^ in which the authors predicted strength of basalt fibre-reinforced geopolymer SCC. The study consisted of both experimental investigation and modelling using ANN for structural assessment of concrete beams. The developed model predicted the ultimate moment capacity of beams with 99% accuracy. Also, Saha et al.^67^ utilized ANN and multivariate regression analysis (MRA) algorithms to predict CS of SCC reinforced with different fibres including glass and polypropylene fibres. The author assessed the accuracy of the algorithms based on correlation coefficient and concluded that ANN can predict CS with a correlation of 0.9919 between actual and predicted values and ANN is more accurate having low RMSE than RMA. Also, Pakzad et al.^21^ predicted CS of SCC modified with steel fibres using a data set of 176 instances. The study used several ML algorithms including ANN, convolution neural networks (CNN), support vector regression (SVR) etc. The models were developed having 9 input variables and only one output variable. The study concluded that CNN is the most robust algorithm having ${\text{R}}^{2}=0.928$ on the test data set. Similarly, another study^68^ incorporated crumb rubber having size between 0.5 and 3.5 mm in place of sand in SCC containing silica fume blended with cement. The study used 63 data points and predicted CS, flexural strength, and elastic modulus by using multivariate regression algorithm. The analysis revealed the excellent performance of the ML algorithm in predicting various F-SCC properties having ${\text{R}}^{2}$ for CS equal to 0.931 and ${\text{R}}^{2}$ equal to 0.937 and 0.992 for flexural strength and modulus of elasticity respectively. This shows the potential of ML techniques to accurately model different SCC properties. The summary of the related literature is represented in Table 1.

**Table 1:** Summary of previous related literature.

S. no.	Algorithm used	Year	Waste materials used	Output(s)	Reference
1	ANN	2016	Fly ash, Slag, Silica fume, RHA, Limestone	CS	^10^
2	DT, XGB, Light Gradient Boosting	2023	Nano silica, Limestone, Fly ash, Marble powder	CS	^69^
3	ANN	2019	Fly ash, Slag, Silica fume	CS	^9^
4	ANN	2017	Fly ash	CS	^70^
5	ANN	2011	Fly ash	CS	^71^
6	Intelligent rule-based enhanced multiclass, SVM	2019	Fly ash	CS	^72^
7	SVR, Deep Learning	2021	Fly ash	CS, Splitting tensile strength	^73^
8	SVM	2020	Fly ash	L-box test, Slump test, V-funnel test, CS	^74^
9	Multivariate adaptive regression spline	2018	Fly ash	Slump test, V-funnel test, L-box test, CS	^75^
10	ANN	2017	Fly ash	CS, Slump flow	^76^
11	ANN	2011	Fly ash	CS	^71^
12	SVR	2023	Fly ash	CS	^77^
13	GEP	2009	Fly ash	Slump flow, CS, J Ring	^78^
14	Multivariate Regression (MVR)	2020	Silica fume, Crumb rubber	Flexural Strength, CS, Modulus of Elasticity	^79^
15	Extreme Learning Machine, long short-term memory (LSTM)	2021	Slag, Fly ash, Silica fume	Slump flow, J Ring	^80^
16	Multilayer perceptron network (MLP), KNN	2022	Fly ash, Slag	CS	^81^

## Research significance

It is evident from Section “Literature review” that ML models like ANN, SVM, RF, and XGB etc. have been frequently utilized to predict different properties of SCC. However, the subject of utilization of ML algorithms like MEP and GEP for C–S prediction of FR-SCC is comparatively unexplored. Thus, this study presents a novel approach to estimate C–S of silica fume-based FR-SCC using MEP and GEP algorithms. The motive behind using MEP and GEP in this study instead of other algorithms like XGB and ANN etc. stems from the fact that these algorithms are regarded as grey-box models in comparison to black-box models like ANN and XGB etc. Although ANN and other algorithms are very famous for their accuracy and robustness, these techniques require the specification of a best model architecture prior to the prediction process and require a large memory^82^. Also, the large number of hidden layers used in these algorithms (as in case of ANN) makes it almost impossible to develop an empirical relationship between input and output variables. Thus, these algorithms are called “black box” models due to the following reasons^83^:

- Lack of transparency.
- Inability to accurately describe the underlying prediction process.
- Inability to create empirical equations relating input and output parameters.

In contrast, MEP and GEP are often referred to as grey-box models^84^ due to the symbolic representation of the underlying prediction process and their ability to express the output as an empirical equation^85^. Also, these techniques do not require any pre-defined optimization of the model architecture, thus requiring less memory and computing power^86^. Therefore, this study is attributed to predicting C–S of FR-SCC using MEP and GEP.

Moreover, despite the fact that FR-SCC offers various advantages over normal concrete, there is a lack of work focusing on the prediction of FR-SCC strength particularly using the two subtypes of GP used in this study called GEP and MEP. Thus, this study is conducted to foster the use of FR-SCC in the industry by providing empirical equations for estimation of its C–S by using MEP and GEP in a comparative manner. The accuracy of models will be compared by using different error metrices and performance indices. The models will also be checked by some external validation criteria and their performance will be compared with regression models too.

## Research methodology

After the problem identification, dataset was compiled to be used for training of the algorithms. This section highlights the working mechanism of both MEP and GEP algorithms and also sheds light on the database collection which was used for training the algorithms. The results of several statistical analyses performed on the gathered database are also represented in Section “Data collection”.

### Prediction models

The two prediction models used in this study are the subtypes of Genetic Programming (GP). GP refers to a type of ML algorithms that use evolutionary rules to create and refine computer programs for solving a problem. Darwin’s principles of natural selection are the basis of genetic programming, and it uses an iterative process to improve and generate increasingly complex programs^87^. The process starts with the formation of a population of programs according to some predefined rules and parameters. These programs are then evaluated using a fitness functions chosen earlier. After the evaluation, the best performing programs are selected to develop new programs. This process repeats over several generations with the goal of reaching to a reliable, accurate and efficient program^78^.

One of the main advantages of GP is that it can discover solutions to problems that humans could not solve. It can find solutions at a much higher pace than humans. This ability makes it an attractive option for solving problems that need a lot of computing power. However, there are certain limitations to it as well. The selection of various fitting parameters of GP for a particular problem maybe challenging and the solution may always not converge. Moreover, it may require a large computing power to find solutions to some problems making it not the most efficient way to find solutions in some cases^88^. But despite these limitations, GP has proven to be a useful tool to solve a wide range of problems in fields like finance, engineering, biology etc. The explanation of two subtypes of GP used in this study is given below:

#### Gene expression programming (GEP)

**Figure 2:**
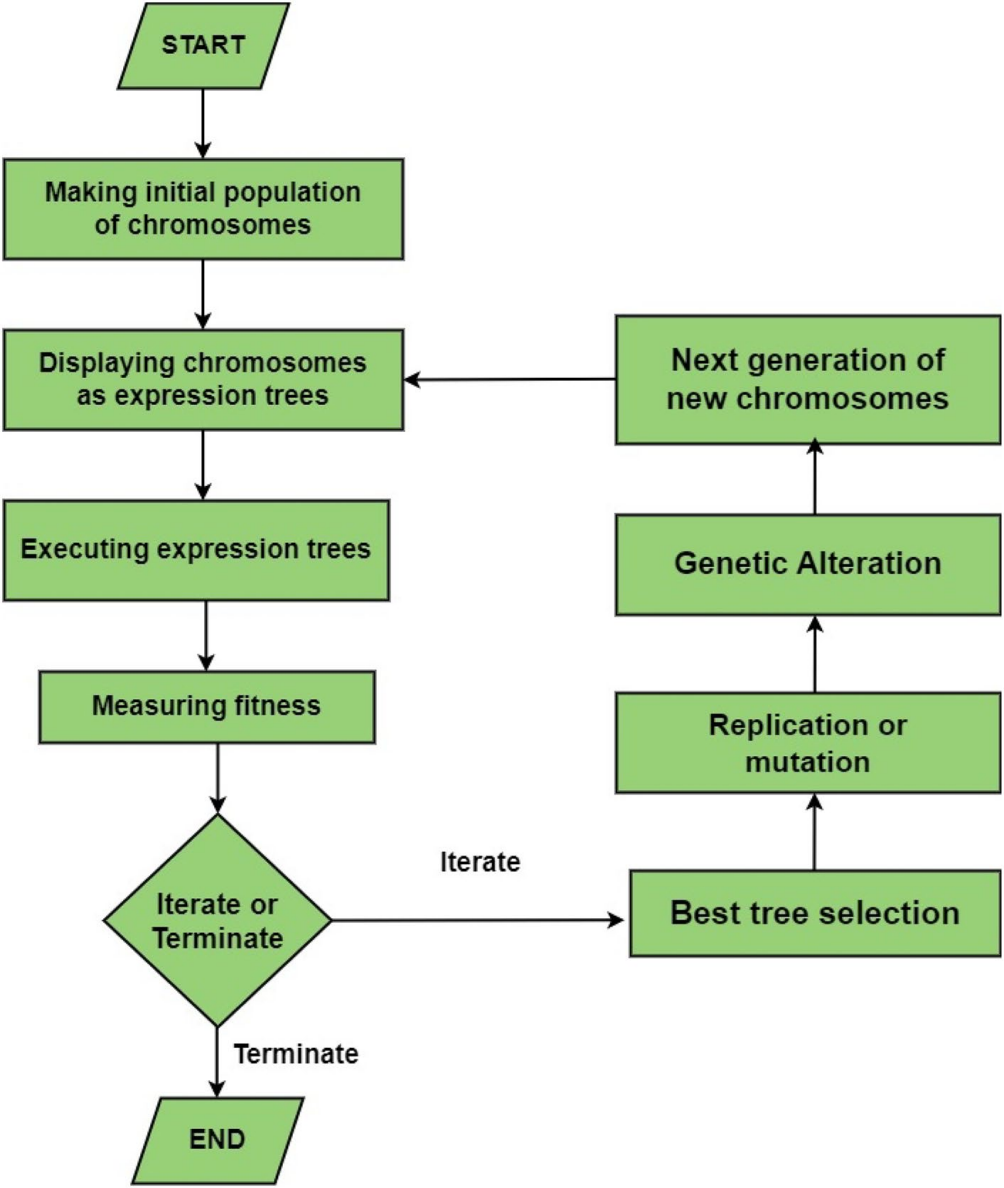
Flowchart of gene expression programming.

GEP is a subtype of genetic programming used to solve problems by evolving a set of mathematical expressions. The central idea of GEP revolves around using a set of genes which represents a small piece of code or function. The genes are built using a set of function called primitive functions. These functions can be simple arithmetic operations like addition, subtraction etc. or more complex mathematical functions such as sine or cosine etc. These genes are combined using various evolutionary rules to create a complete program^89^. This process of creating the programs by combining different genes is similar to that in human beings. That’s why it is called gene expression programming. The process of solving a problem using GEP begins by creating a random population of programs called chromosomes. Then these chromosomes are evaluated using a “fitness function”. The good performing chromosomes are selected for the next generation while the worst performing ones are discarded^90^. This process repeats over several generations with the goal of having a population of chromosomes that gives the highest accuracy. The algorithm constantly modifies and improves the chromosomes while running eventually coming at a solution as close to the actual as possible^91^. The flowchart of GEP algorithm is shown in Fig. 2.

#### Multi expression programming (MEP)

**Figure 3:**
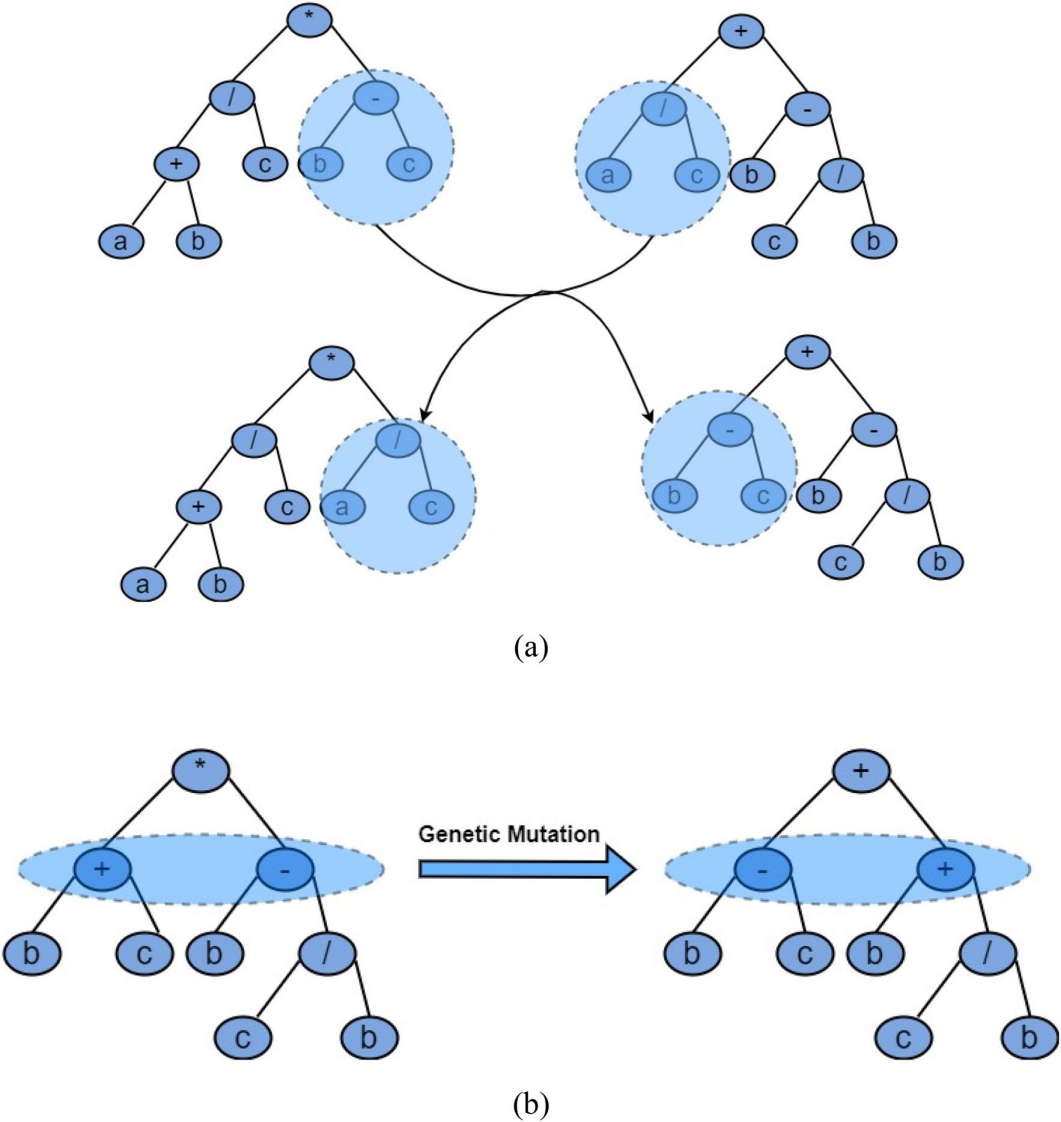
Representation of (**a**) genetic crossover, (**b**) genetic mutation.

**Figure 4:**
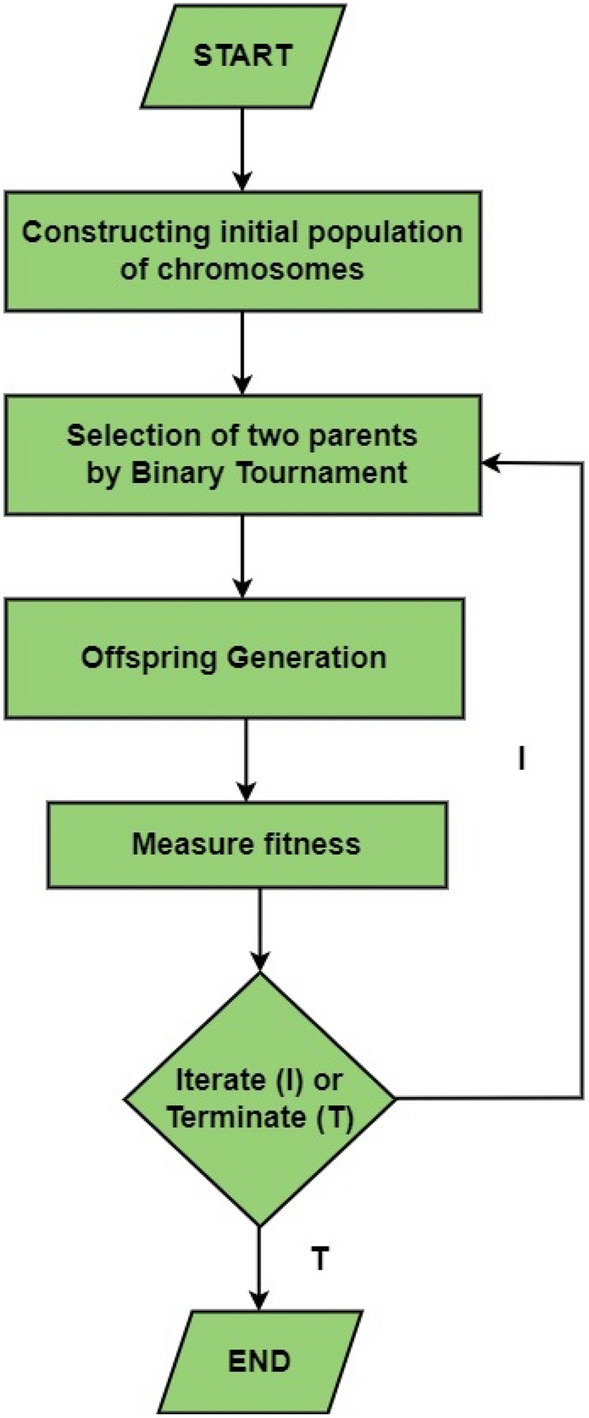
Flowchart of multi expression programming.

MEP is a sub technique of GP developed by Oltean^92^. It is an optimization method that involves producing and evolving a set of mathematical expressions to find solution of a problem^93^. MEP offers the advantage of handling a wide variety of problems whether non-linear, multimodal or they have complex constraints. This ability of MEP stems from the fact that it does not need any previous assumptions about the problem on hand, rather it creates a population of mathematical expressions and chooses the best performing expressions to be the solution of the problem using various evolutionary rules^94^. The algorithm starts by creating a set of randomly generated initial expressions. These expressions are then evaluated according to their ability to solve the problem accurately. The expression which performed good are used to develop a new population of expressions. The new population of expressions is developed using evolutionary principles such as mutation and crossover on the initially developed expressions. Crossover means combining two expressions to create a new one while mutation involves altering an existing expression^95^. The expression tree representation of both mutation and crossover is given in Fig. 3. Thus, MEP explores new solutions to the problem and discover potentially better expressions for the representation of the optimization problem through these two processes. This whole process of generating expressions, evaluating them, and using them to create a new population is called an iteration. After the creation of a new generation of expressions, the process continues until the desire accuracy is reached or a predefined criteria is met. This criterion either be a certain fitness value or a specific number of iterations. The flowchart of MEP algorithm is shown in Fig. 4.

### Data collection

The collection of a reliable dataset is the central point of developing a machine learning model. Thus, to develop robust models, a reliable database of 78 points is collected from the internationally published literature given in Table A. Although this dataset is a good starting point to be used for developing predictive models, it is recommended that future studies should consider much larger datasets so that the algorithms can be trained and tested on a variety of data and the resulting models can be used on variable data configurations and distributions. For the selection of most influential parameters to predict C–S, several initial trials were performed, and ultimately the following six input parameters were chosen: water-to-cement ratio, silica fume, coarse aggregate, fine aggregate, fibres, and superplasticizer. These six parameters will be used to predict the single output i.e., C–S. To develop a robust ML model, the data should be splitted in two or three sets^96–98^. Thus, the dataset gathered is split into two parts named training and validation data. Training data (70%) is used to train the model and validation set (30%) is used to test the model’s accuracy. This splitting makes sure that the model is not overfitted to the training data and is equally accurate for predicting on the validation data^99^. The statistical description of the variables used to develop the models, along with their units and symbols is represented in Table 2. Notice from the minimum and maximum values of explanatory variables that the dataset is spread over a large range. The spread of data over a large range ensures a more robust and accurate model^100^.

**Table 2:** Description of the dataset used for model development.

	Units	Symbol	Maximum	Minimum	Average	Standard deviation
W/C ratio	–	${x}_{0}$	0.739	0.39	0.52	0.125
Silica fume	kg	${x}_{1}$	978	840	913.7	44.62
Coarse aggregate	kg	${x}_{2}$	870	707	811.29	35.97
Fine aggregate	kg	${x}_{3}$	190	70	120.35	40.74
Fiber	kg	${x}_{4}$	1.40	0	0.730	0.57
Superplasticizer	kg	${x}_{5}$	14.7	7	9.47	1.87
Strength	MPa	${f}_{c}{\prime}$	68.5	28.24	48.10	12.9

**Figure 5:**
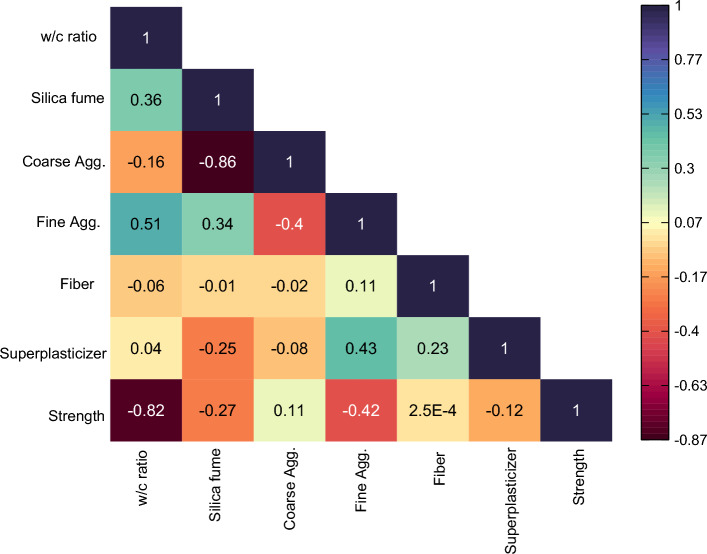
Correlation matrix of variables.

**Figure 6:**
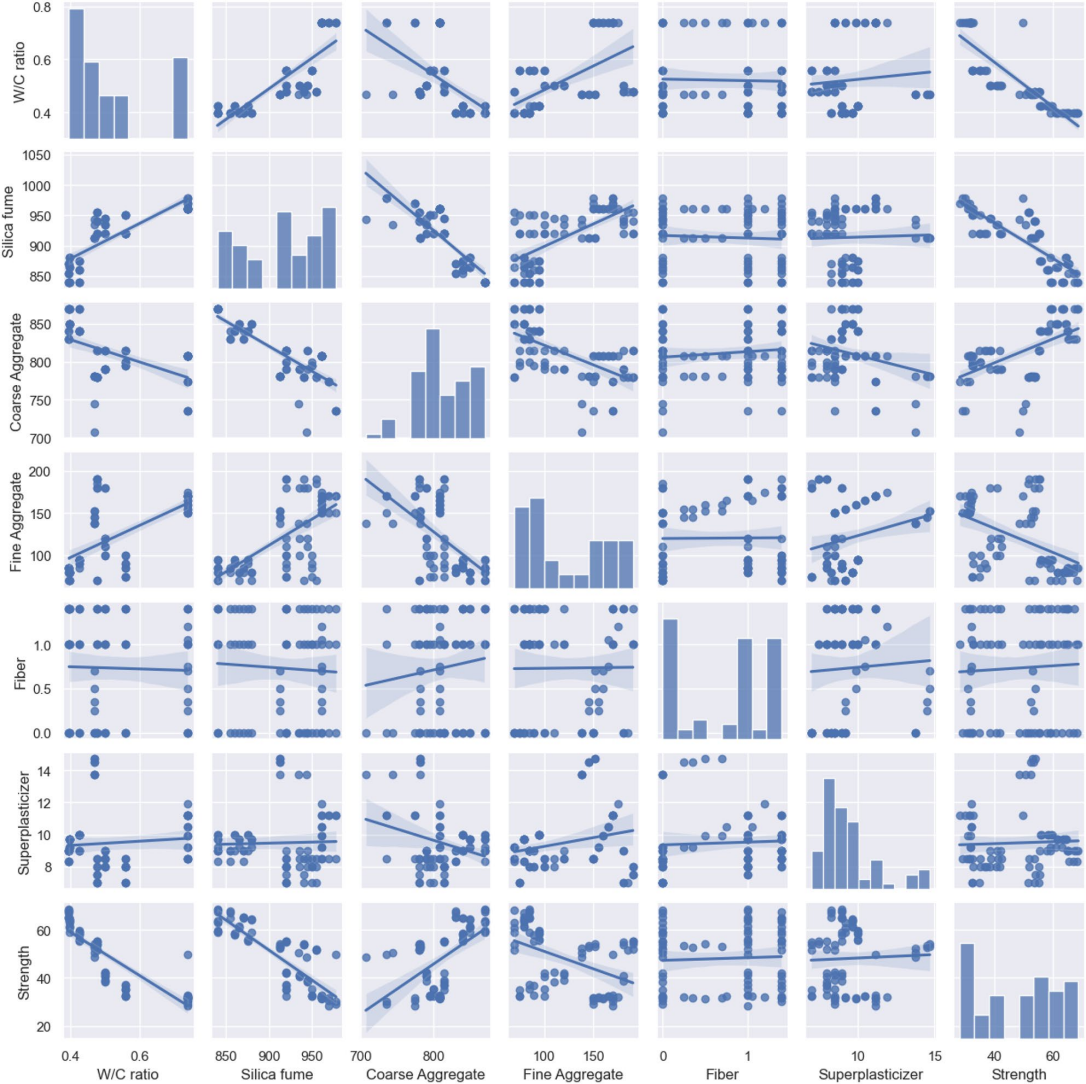
Scatter matrix of variables and their frequency distribution.

The interdependence between various variables used to develop the models can be checked using two very useful statistical analysis techniques called correlation matrix and scatter matrix shown in Figs. 5 and 6 respectively. A correlation matrix is used to investigate the effect of explanatory variables on each other. It quantifies the relationship between different variables used in the study in the form of coefficient of correlation (R). By looking at the R values between two variables, we can tell the extent to which these variables are related and what effect does the change in one variable have on the other variable. The correlation between any two variables can be positive, negative or zero. Generally, a value above 0.8 indicates the presence of a good correlation between two variables^101^. The interdependence between multiple variables must be checked before developing a model. If most of the variables are strongly correlated to each other, then it might cause complications in the model. This is sometimes referred to as multi-collinearity^102^. Notice from Fig. 5 that strength is affected by all the explanatory variables used in the study. It is strongly correlated with cement and is having the weakest correlation with the fibre content. The correlations between other variables are less than 0.8 mostly so they will not give rise to the problem of multi-collinearity.

Scatter matrix is another tool used widely alongside the correlation matrix. Its main function is to help visualize the bivariate correlations between all the possible combinations of the variables used in the model. It offers useful insights and helps to visualize different combinations of regressions of the variables used^103^. Notice from Fig. 6 that the x-axis and y-axis of scatter matrix are associated with the variables used in the study. It’s a grid of different scatter plots and each scatter plot helps to visualize and explore the relationship between any two variables. It can also be used as a useful tool for outlier detection^104^. The frequency distribution histograms of all variables used to build the models are also shown in the diagonal of the scatter matrix. It is recommended to have the distribution of variables as close to the normal distribution as possible since it helps in the development of a reliable model that covers a wide range of variables^105^. Thus, it is useful tool that depicts the regression between variables, distribution of variables and helps in outlier detection simultaneously.

### Performance assessment

The performance of both models will be checked using some statistical error metrices suggested in the literature. These error metrices include mean absolute error (MAE), root mean square error (RMSE), coefficient of determination (${\text{R}}^{2}$) and performance index (p) etc. The mathematical expressions to calculate these metrices are given below:

$$\text{Mean Absolute Error }(\text{MAE}) =\frac{\Sigma |\text{x }-\text{ y}|}{N}$$

$$\text{Coefficient of Determination }({\text{R}}^{2}) =1-\frac{\sum {\left(x-y\right)}^{2}}{\sum {\left(y-{y}_{mean}\right)}^{2}}$$

$$\text{Root Mean Square Error }(\text{RMSE}) =\sqrt{\frac{{\sum (\text{x }-\text{ y})}^{2}}{N}}$$

$$\text{a}20 -\text{ index }=\frac{N20}{N}$$

$$\text{Performance Index }(\text{p}) =\frac{RRMSE}{1+R}$$

$$\text{Objective Function }(\text{OF}) =\left(\frac{{N}_{Training}-{N}_{Validation}}{N}\right){p}_{Training}+2(\frac{{N}_{Validation}}{N}){p}_{Validation}$$

where x and y represent actual and predicted C–S values while N and $N20$ represents the total samples and number of samples for which the ratio of actual and predicted values lies between 0.80 and 1.20 respectively.

An accurate and reliable model should have a higher correlation between actual and predicted values. This correlation is quantified by the coefficient of determination (${\text{R}}^{2}$). The ${\text{R}}^{2}$ value ranges from 0 to 1, and value above 0.8 shows an excellent correlation between the actual and predicted values^106^. However, ${\text{R}}^{2}$ alone cannot be used as an indication of accuracy of the model. It is due to the inability of ${\text{R}}^{2}$ to respond to the output being multiplied or divided with a constant^107^. Thus, the error metrices such as MAE, RMSE have also been identified as crucial for evaluating the efficiency of any machine learning model^108^. MAE and RMSE both signify the value of average error. RMSE gives more weight to large errors, thus it is used for interpretation of large errors. According to Despotovic et al.^109^, a model is outstanding if RMSE value lies between 0 and 0.11. MAE on the other hand, associates less weight with larger errors and its value is always less than RMSE. It is used as an indication of the average error between the experimental and predicted values in the dataset. The newly developed a20-index is another useful metric to evaluate the performance of ML algorithms. It signifies the number of samples with deviation more than ± 20% from the experimental values^38^. The ideal value of a20-index is 1 and a model must have value of a20 close to 1 to be acceptable^110^.The metrices OF and ƿ can have values between 0 and infinity but a good model must have ƿ and OF value less than 0.2^111^. Performance index offers the advantage of covering both the values of R and relative root mean square error (RRMSE) at the same time. Similarly, OF takes into account the effect of R, RRMSE and the number of data points in training and validation sets. So, it is an indicator of the overall performance of the model. Thus, a model with lower OF indicates overall superior performance. The same criteria were used during trials of model development and the models represented in the study are those that gave the least OF value.

## Results and discussions

### Formulization of strength using GEP

A specialized software called GeneXpro Tools version 5.0 was used to employ the GEP algorithm in current study. Before training, the database was split randomly into training and testing sets in 70:30 ratio as specified earlier using the data split option in the software. Also, there are several fitting parameters of GEP algorithm that need to be specified before the commencement of actual model training. This is because each algorithm calls for a specific set of hyperparameters which can only be determined by extensive testing using different set of values. For GEP algorithm, the important parameters include number of genes, head size, functions, number of chromosomes etc. The selection right set of values for these parameters is important since the model’s performance is affected by them. For example, head size and number of chromosomes dictate the convergence of the model towards the solution. The GEP parameters used in this study were chosen after extensive testing and after consulting previous studies. The initial parameter values were selected using a previous literature recommendation^1,112^ and these values were varied across a wide range of values. The set of values which yielded highest accuracy were used to build the GEP models for C–S prediction in current study are given in Table 3. Notice from Table 3 that the number of genes are selected as 4 which indicates that there will be 4 sub-expression trees generated for C–S prediction. These sub-expression trees will be linked by the specified linking function (addition in our case) to get the final equation for output prediction. Similarly, the head size and number of chromosomes were carefully chosen to not to overfit the model or make it computationally challenging because increasing these parameters beyond a certain point increases the run time of the algorithm and complexity of the model. Also, notice that the set of functions used to be included in the final equation for C–S prediction consist of simple arithmetic functions and square root function. It was deliberately done in order to keep the resultant equations simple and compact for fast computation and implementation.

**Table 3:** Hyperparameters of GEP and MEP model.

Parameters	Settings
GEP parameters	
No. of chromosomes	20
No. of genes	4
Head size	10
Linking function	Addition
Constants per gene	10
Functions	+, −, ×, ÷, sqrt
MEP parameters	
No. of subpopulations	200
Subpopulation size	500
Code length	40
Crossover probability	0.9
Number of generations	500
Runs	10
Functions	+, −, ×, ÷, sqrt

The size of the population controls the running duration of the program. The model generation was initiated by selecting the number of chromosomes equal to 10. The number of chromosomes and other hyperparameters are varied until the equation with highest accuracy is reached with chromosome number at 20 and head size equal to 4 and 10 respectively. The resulting equation contains the explanatory variables mentioned in Eq. (1).

1 $${f}_{c}{\prime}= ({x}_{0},{x}_{1},{x}_{2},{x}_{3},{x}_{4},{x}_{5})$$

**Figure 7:**
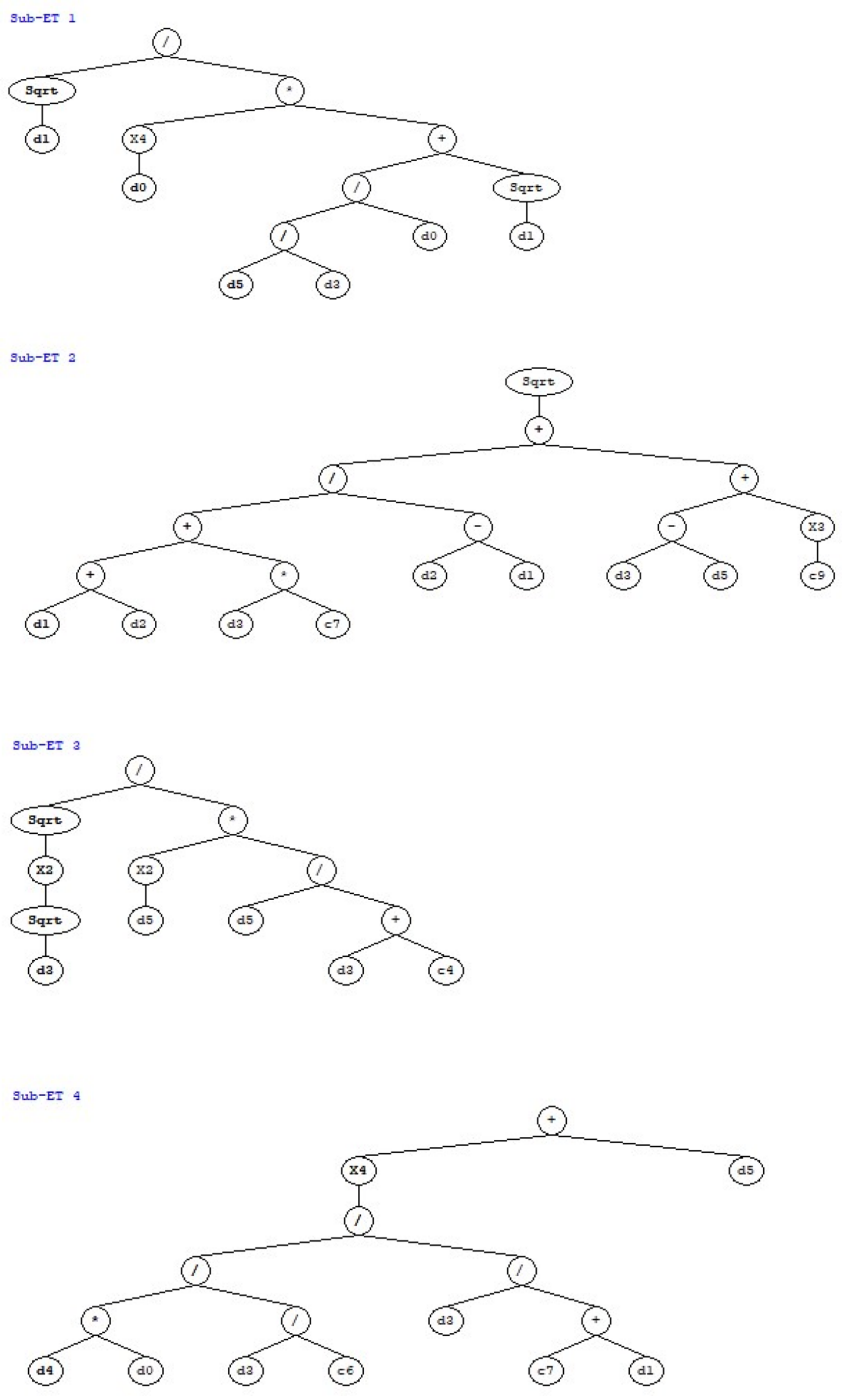
Expression tree representation of GEP equation.

The addition function is used as a linking function for GEP model with other arithmetic operation such as multiplication, subtraction, division etc. These functions are chosen to keep the resulting equation simple. The output of GEP algorithm given in the form of expression tree represented in Fig. 7 is decoded to get Eq. (2). The subexpressions from each expression tree are linked by the chosen linking function to get the final equation.

2 $${f}_{c}{\prime}=A+B+C+D$$

where:

$$A= \frac{\sqrt{{x}_{1}}}{{\left({x}_{0}\right)}^{4}\{\left(\frac{{x}_{0}{x}_{5}}{{x}_{3}}\right)+\sqrt{{x}_{1}} \}}$$

$$B= \sqrt{\left(\frac{\left({x}_{1}+{x}_{2}\right)-9.306{x}_{3}}{{x}_{2}-{x}_{1}}\right)+(\left({x}_{3}-{x}_{5}\right)+143.38})$$

$$C= \frac{\sqrt{{(\sqrt{{x}_{3}})}^{2}}}{{\left({x}_{5}\right)}^{2}(\frac{{x}_{5}}{{x}_{3}+15.45})}$$

$$D= {(\frac{\frac{({x}_{0}{x}_{4})}{-0.137}}{\frac{{x}_{3}}{{x}_{1}+827.31}})}^{4}+{x}_{5}$$

### Statistical assessment of GEP

**Figure 8:**
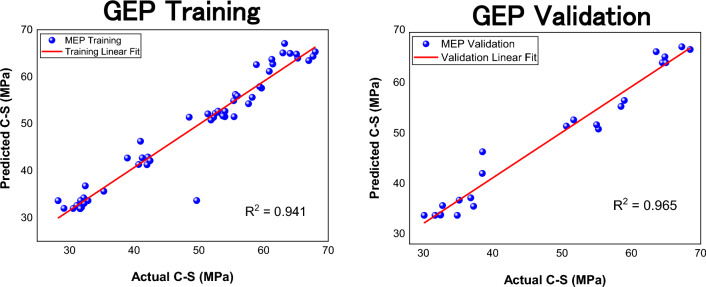
Scatter plot of GEP.

**Figure 9:**
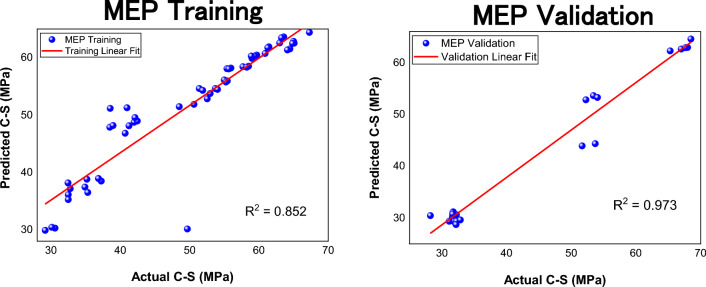
Scatter plot of MEP.

The developed models are assessed by calculating the statistical metrices for both data sets. The summary of error metrices of both models is given in Table 4. Notice from the table that the ${\text{R}}^{2}$ value of both models is well above the threshold of 0.8. It can also be seen from the scatter plots given in Figs. 8 and 9. Also, the ƿ values are significantly below 0.2 which means that there exists a strong correlation between actual and model predicted values and both models can efficiently predict strength of FR- SCC. Notice that the training MAE of GEP is less than MEP, which implies that GEP did a good job in optimizing to the training data than MEP. Also, the RMSE values are lower for GEP. This is because GEP has lesser predictions with large errors as compared to MEP. It is also indicated by higher a20-index values of GEP. The validation value of a20-index is 1 which means that GEP does not give a single prediction that deviates more than ± 20% from the experimental values. Moreover, the value of objective function which is a useful metric to assess the overall performance of the model is lower for GEP. Thus, it can be inferred that GEP despite having slightly lesser validation ${\text{R}}^{2}$ than MEP, proved to be more robust than MEP.

**Table 4:** Error metrices of both models.

	GEP		MEP	
	Training	Validation	Training	Validation
MAE	1.97	2.11	3.02	2.75
RMSE	3.06	2.69	4.74	3.58
${\text{R}}^{2}$	0.941	0.965	0.852	0.973
ƿ	0.032	0.028	0.049	0.040
a20-index	0.981	1	0.945	0.956
OF	0.0296		0.031

### Formulization of strength using MEP

The MEP model development was done using a software known as MEPX 2021.05.18.0. Same as for GEP algorithm, there are several parameter settings that must be done before actual development of a predictive model by MEP. The most important MEP parameters include subpopulation size, number of subpopulations, set of functions, code length etc. The initial values of these parameters were chosen using recommendations from prior studies^113–115^. After having some preliminary values, these parameters were varied across a wide range of values and a trial-and-error approach was followed to reach at the set of parameters which resulted in maximum accuracy of the predictive models. The set of these hyperparameter values which exhibited highest accuracy for C–S prediction are given in Table 3. The number of computer programs developed by the algorithm are dictated by the size of subpopulation. A predictive model having large size of subpopulations can be more accurate and reliable, however it can also be computationally challenging, complex, and time-consuming^116^. Similarly, number of generations are used to indicate the iterations done by the algorithm before it is terminated. Its value was selected as 500 in current study for development of both models so that the algorithm performs a large number of iterations of generating a suitable chromosome for representation of the solution. In the same way, the parameter code length is directly related with the length of the resulting MEP equation. An abnormally larger value of code length may result in the equation being too complex and computationally exhaustive while a smaller value may result in premature convergence of the algorithm and lack of accuracy. Therefore, its value was also chosen carefully by consulting previous studies and considering many possible values before reaching at the values given in Table 3^117^. Moreover, the set of functions to construct the MEP equations involve simple mathematical functions as an attempt to keep the equations simple.

The model development by MEP is an iterative and evolving process trying to optimize the accuracy of the model and complexity of the resulting equation. To find an optimal mode, several initial trials were performed until reaching at one final equation that incorporates the effect of all of the variables and gives the maximum accuracy. The expression given by MEP is a function of the following input parameters:

3 $${f}_{c}{\prime}= ({x}_{0},{x}_{1},{x}_{2},{x}_{3},{x}_{4},{x}_{5})$$

The output of the MEP algorithm is given in the form a C++ code. It is decoded to get the expression given by Eq. (4). Notice that the equation is generated by using simple mathematical operations such as addition, subtraction, division, multiplication, and square root.

4 $${f}_{c}{\prime}= \frac{\sqrt{{x}_{2}}}{{x}_{0}}-{(x}_{0}{x}_{4})-(\frac{2{x}_{1}-{x}_{2}-\frac{{x}_{0}}{{x}_{5}}}{{x}_{3}})$$

### Statistical assessment of MEP

**Figure 10:**
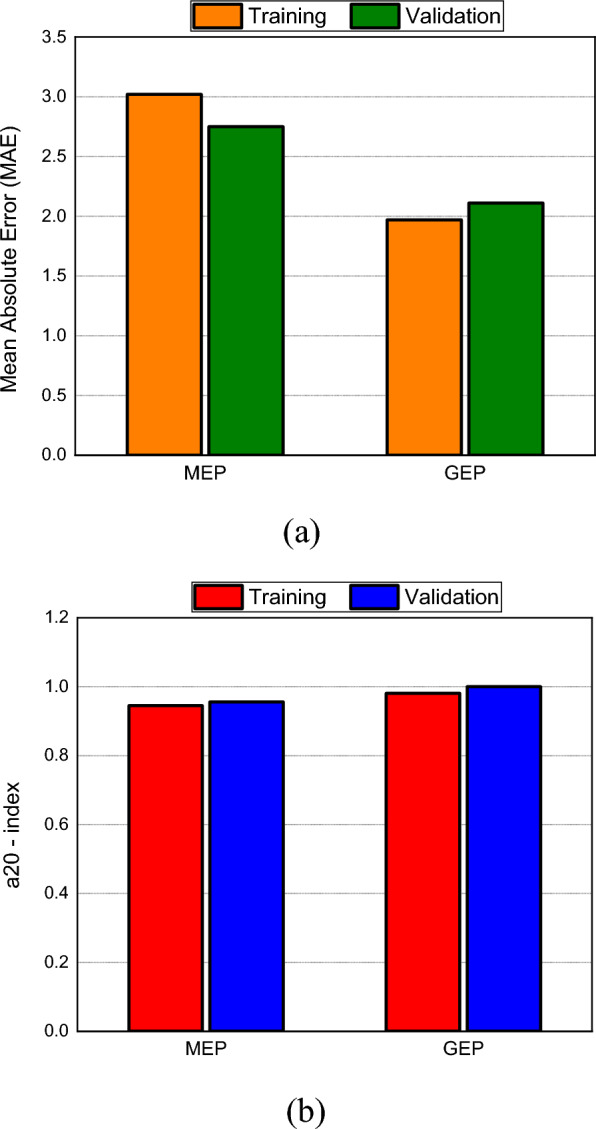
Errors comparison of both models; (**a**) MAE; (**b**) a20-index.

The error metrices for MEP are given in Table 4. A comparison has been made between training and validation error metrices of both models and results are shown in Fig. 10. Notice from Fig. 10 (a) that the training MAE value of GEP is less than MEP which means that there is less average deviation in GEP values from actual values. Also, the validation RMSE value of GEP is less than MEP which means MEP has more predictions with larger errors than GEP. It is also clear from training and validation a20 index of MEP. The validation performance index is closer to zero for GEP than MEP, and the value of objective function is also less for GEP. Thus, it is clear that MEP has more deviations in its predictions than GEP and it can be concluded that GEP did a good job in formulating an empirical equation based on the given data that relates all the input variables with the output with great accuracy. Although, the GEP equation is a bit complex and requires more computing effort than MEP equation, its performance overall is better than MEP equation as indicated by objective function and a20-index values. Thus, GEP equation will be considered for further analysis.

### External validation of MEP and GEP models

Several external testing criteria are recommended by previous studies to test the developed models. The summary of these validation checks along with their recommended range in the literature is given in Table 4. The inclination of the regression lines passing over the origin is depicted by the value of s or ${s}{\prime}$. For a model to be accurate, both values must be close to 1. Similarly, the squared coefficient ${R}_{o}^{2}$ and ${R}_{o}^{{\prime}2}$ should also be close to 1 for an accurate model^85^. The values of MEP and GEP equations for these validation checks are also shown in Table 5. Notice from the table that both the models satisfy the external validation checks, and their values lie in the recommended range for both models. This means the equations developed by MEP and GEP will accurately predict C–S of FR-SCC.

**Table 5:** External validation of MEP and GEP model.

Expression	Criteria	MEP	GEP	Reference
$s=\frac{\sum_{i=1}^{n}({e}_{i}\times {m}_{i})}{\sum_{i=1}^{n}({e}_{i}^{2})}$	0.85 < s < 1.15	0.99	0.99	^118^
${s}{\prime}=\frac{\sum_{i=1}^{n}({e}_{i}\times {m}_{i})}{\sum_{i=1}^{n}({m}_{i}^{2})}$	0.85 < ${s}{\prime}$ < 1.15	0.99	0.99	^118^
${R}_{m}= {R}^{2}\times (1-\sqrt{|{R}^{2}-{R}_{o}^{2}|})$ where	${R}_{m}>0.5$	0.515	0.55	^119^
${R}_{o}^{2}=1-\frac{\sum_{i=1}^{n}{\left({m}_{i}-{e}_{i}^{o}\right)}^{2}}{\sum_{i=1}^{n}{\left({m}_{i}-{m}_{i}^{o}\right)}^{2}}$*,*${e}_{i}^{o}=s\times {m}_{i}$	${R}_{o}^{2}\approx 1$	0.98	0.99	^120^
${R}_{o}^{{\prime}2}=1-\frac{\sum_{i=1}^{n}{({e}_{i}-{m}_{i}^{o})}^{2}}{\sum_{i=1}^{n}{({m}_{i}-{m}_{i}^{o})}^{2}}$*,*${m}_{i}^{o}={s}{\prime}\times {e}_{i}$	${R}_{o}^{{\prime}2}\approx 1$	0.99	0.99	^121^

### Comparison with linear and non-linear regression models

**Figure 11:**
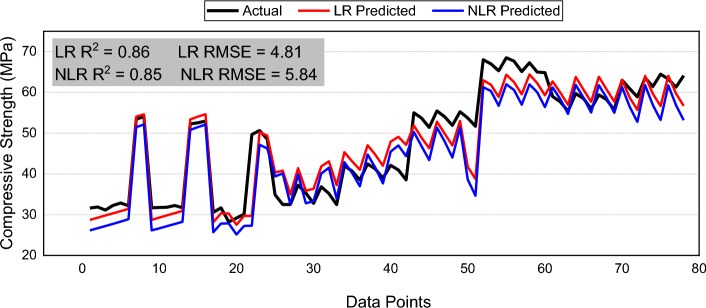
Series plot of actual, linear, and non-linear regression predictions.

This study used six explanatory variables for strength prediction of FR-SCC. To date, no empirical equation has been devised using GEP or MEP for strength estimation of FR-SCC that considers the same set of input variables. So, it is necessary to compare the results of MEP and GEP with Linear Regression (LR) and simple Non-linear Regression (NLR) models developed with the same set of input variables using the same dataset. Thus, the LR and NLR equations for calculating C–S of FR-SCC are given by Eqs. (5) and (6). It is evident from the series plot between actual, NLR and LR predicted values given in Fig. 11 that LR and NLR does not perform well on the dataset. The average error of LR and NLR is 3.2 and 4.2 respectively compared to 2.11 of GEP and 2.75 of MEP. Similarly, the RMSE value of LR and NLR are greater than that of MEP and GEP. Also, NLR predicted values have a lesser correlation with actual values and it failed to accurately predict strength at several points and gave predictions with average error as large as 25. Also, LR and NLR have some other disadvantages as well. The simple regression models assume some pre-defined equations and normality of the residuals for making predictions^122^. Thus, simple LR and NLR are not suitable for modelling the relationship between strength and the input variables used in this study. This further reinforces the importance of using machine learning techniques like MEP and GEP to capture the complex multi non-linear relationships between input and output variables.

5 $${f}_{c}{\prime}=270.55-51.2{x}_{0}-0.2{x}_{1}-0.03{x}_{2}+0.07{x}_{3}+0.14{x}_{4}+0.23{x}_{5}$$

6 $${f}_{c}{\prime}=187.8-34{{x}_{0}}^{2}-0.00014{{x}_{1}}^{2}-\left(3.2\times {10}^{-5}\right){{x}_{2}}^{2}+0.0003{{x}_{3}}^{2}-0.04{{x}_{4}}^{2}+0.005{{x}_{5}}^{2}$$

### Best model selection

**Figure 12:**
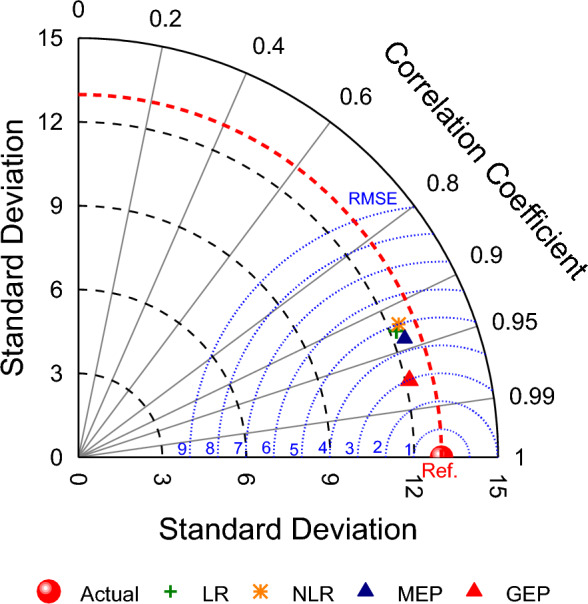
Taylor diagram to compare model and regression predicted values.

Based on the above discussion, LR and simple NLR displayed less accuracy as compared to MEP and GEP. Out of the two evolutionary techniques used, GEP performed better than MEP. The comparison between actual, algorithm prediction and regression predicted values can be well understood by a Taylor Diagram^123^ as shown in Fig. 12. It offers the significant advantage of simultaneously using RMSE, coefficient of correlation and standard deviation of the predicted values to draw comparison between different models. The accuracy of various models is checked by computing their distance from the target point of experimental data. It can be seen from taylor diagram that GEP performs well in terms of RMSE and correlation, and it is closer to the reference data line. In contrast, the MEP shows larger RMSE and lesser correlation and is placed away from reference line. Moreover, LR and simple NLR has lowest correlation and largest RMSE and are located farther away from actual data. Hence, GEP comes turns out to be the most accurate followed by MEP. Thus, the overall order of model accuracy is: GEP > MEP > LR > NLR. Therefore, the sensitivity analysis will be performed on GEP equation as it is more accurate and have lesser errors than MEP, LR and NLR.

### Overfitting of models

A common problem that can arise when dealing with mathematical modelling is overfitting. It happens when an algorithm fits well to the data it is trained on but cannot maintain its accuracy when exposed to new data^124^. It is important to assess a model against the possible issue of overfitting. A model can be checked for overfitting by drawing a comparison between the error metrices of its training and validation data^125^. If a model gives predictions with large errors on validation data, it implies that the model overfitted to the training data and is not suitable for unseen data and the model is not a generalized one. Regarding the models developed in the current study, it is evident from Table 4 and Fig. 9 that the validation error metrices for both models doesn’t significantly vary from the training ones. It means that the model maintains its accuracy when it is used for making predictions on new data. Therefore, the developed equations by MEP and GEP have good generalization capabilities and can be practically used to forecast C–S of FR-SCC.

### Sensitivity analysis (SA)

**Figure 13:**
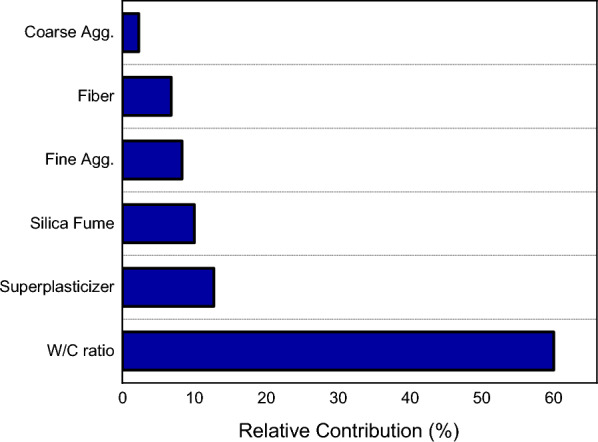
Relative contribution of input variables.

Since the GEP model performed well than its counterpart, sensitivity analysis is performed on the GEP equation to check the impact of input variables on output. SA tells about the sensitivity of the output to the uncertainties in the input data. Higher SA value of a variable, higher the effect it has on the output^96^. It is done on the GEP mode using Eqs. (7) and (8). The results of sensitivity analysis are shown in Fig. 13.

7 $${N}_{i}={f}_{max}\left({x}_{i}\right)-{f}_{min}\left({x}_{i}\right)$$

8 $$\text{SA}= \frac{{\text{N}}_{\text{i}}}{\sum_{\text{n}}^{\text{j}=1}{\text{N}}_{\text{j}}}$$

where ${x}_{i}$ = *i t*ℎ input variable with all other variables constant, ${f}_{max}\left({x}_{i}\right)$ = maximum predicted output, ${f}_{min}\left({x}_{i}\right)$ = minimum predicted output.

The results indicate that all input variables contribute to in determine the C–S of FR-SCC. However, water-to-cement ratio is the crucial factor to predict strength (60%), followed by superplasticizer (12%) and silica fume (10%). Other variables like fine aggregate (8.27%), fibres (6.77%) and coarse aggregate (2.26%) have comparatively less contribution in predicting the strength. The result of SA depends upon several factors such as data points used to construct the models and the number of input variables^126^. In relation to this, several studies reported that water-to-cement is most important in prediction of SCC strength^127,128^. It is also evident from the strong correlation between water-to-cement ratio and strength given in Fig. 4. These studies also highlight the relative importance of superplasticizer in the output prediction. Ahmed et al.^129^ reported the less effective role of coarse aggregate towards predicting the strength. Moreover, the lesser contribution of fibres and fine aggregate also aligns with the findings of previous research^41,96,127^.

## Conclusions

This study aimed to provide empirical equations for estimating C–S of FR-SCC using MEP and GEP using a dataset collected from internationally published literature to foster the use of FR-SCC in construction industry. The following conclusions were drawn from this research:

- Both MEP and GEP algorithms expressed their output as an empirical equation for computing C–S and the error evaluation revealed that the performance of both algorithms is satisfactory as per the criteria suggested in the literature.
- The GEP algorithm outperformed MEP having training ${R}^{2}=$ 0.941 compared to 0.85 of MEP. Also, GEP yielded lesser predictions with larger errors as depicted by RMSE value of 2.69 compared to 3.58 of MEP.
- The accuracy of the models was also checked by employing external validation checks and the results indicated that both models are accurate. The comparison of MEP and GEP with LR and NLR models also suggests the same.
- The GEP equation was considered for sensitivity analysis and the results showed that water-to-cement ratio, superplasticizer, and silica fume are more important parameters to predict strength having contributions 60%, 12% and 10% respectively. However, fine aggregate (8.27%), fibres (6.77%) and coarse aggregate (2.26%) have relatively less contribution.

### Supplementary Information

Supplementary Information.

Supplementary Table 1.

## Data Availability

The data used to develop the models is provided in the article.
