# Supplementary material for: Predicting 28-day compressive strength of fibre-reinforced self-compacting concrete (FR-SCC) using MEP and GEP
Source: Sci Rep. 2024 Jul 27;14:17293. doi: 10.1038/s41598-024-65905-5 (PMC11283530; doi:10.1038/s41598-024-65905-5)
Supplement: Supplementary file 1 — Supplementary Information. [file 41598_2024_65905_MOESM1_ESM.docx]

**Appendix.**

**Table A.** Dataset used for model development.
